# The British American Journal of Medical and Physical Science

**Published:** 1845-09

**Authors:** 


### *The British American Journal of Medical and Physical Science.*

The publication of the above-named Journal was commenced in March last, and, from the ability with which it is conducted, has already secured for itself a high rank among the medical periodicals of the day. It is edited by Archibald Hall, M. D., Lecturer on Chemistry in the University of the McGill College, and is published monthly in quarto form; each number contains thirty-two pages. So valuable a collaborator in the field of medical science, will, no doubt, be liberally sustained. We have received the July and August numbers, and should be glad to obtain the preceding ones.

